# Rapid detection of *Mannheimia haemolytica* in lung tissues of sheep and from bacterial culture

**DOI:** 10.14202/vetworld.2015.1073-1077

**Published:** 2015-09-15

**Authors:** Jyoti Kumar, Shivendra Kumar Dixit, Rajiv Kumar

**Affiliations:** 1 Division of Animal Health, Central Sheep and Wool Research Institute, Avikanagar - 304 501, Rajasthan, India; 2 Animal Biotechnology Section, Central Sheep and Wool Research Institute, Avikanagar - 304 501, Rajasthan, India

**Keywords:** lung tissues, *Mannheimia haemolytica*, multiplex polymerase chain reaction, *Pasteurella haemolytica* serotype-1 specific antigens, Rpt2, 12S ribosomal RNA, sheep

## Abstract

**Aim:**

This study was aimed to detect *Mannheimia haemolytica* in lung tissues of sheep and from a bacterial culture.

**Introduction:**

*M. haemolytica* is one of the most important and well-established etiological agents of pneumonia in sheep and other ruminants throughout the world. Accurate diagnosis of *M. haemolytica* primarily relies on bacteriological examination, biochemical characteristics and, biotyping and serotyping of the isolates. In an effort to facilitate rapid *M. haemolytica* detection, polymerase chain reaction assay targeting *Pasteurella haemolytica* serotype-1 specific antigens (PHSSA), Rpt2 and 12S ribosomal RNA (rRNA) genes were used to detect *M. haemolytica* directly from lung tissues and from bacterial culture.

**Materials and Methods:**

A total of 12 archived lung tissues from sheep that died of pneumonia on an organized farm were used. A multiplex polymerase chain reaction (mPCR) based on two-amplicons targeted PHSSA and Rpt2 genes of *M. haemolytica* were used for identification of *M. haemolytica* isolates in culture from the lung samples. All the 12 lung tissue samples were tested for the presence *M. haemolytica* by PHSSA and Rpt2 genes based PCR and its confirmation by sequencing of the amplicons.

**Results:**

All the 12 lung tissue samples tested for the presence of PHSSA and Rpt2 genes of *M. haemolytica* by mPCR were found to be positive. Amplification of 12S rRNA gene fragment as internal amplification control was obtained with each mPCR reaction performed from DNA extracted directly from lung tissue samples. All the *M. haemolytica* were also positive for mPCR. No amplified DNA bands were observed for negative control reactions. All the three nucleotide sequences were deposited in NCBI GenBank (Accession No. KJ534629, KJ534630 and KJ534631). Sequencing of the amplified products revealed the identity of 99-100%, with published sequence of PHSSA and Rpt2 genes of *M. haemolytica* available in the NCBI database. Sheep specific mitochondrial 12S rRNA gene sequence also revealed the identity of 98% with published sequences in the NCBI database.

**Conclusion:**

The present study emphasized the PCR as a valuable tool for rapid detection of *M. haemolytica* in clinical samples from animals. In addition, it offers the opportunity to perform large-scale epidemiological studies regarding the role of *M. haemolytica* in clinical cases of pneumonia and other disease manifestations in sheep and other ruminants, thereby providing the basis for effective preventive strategies.

## Introduction

Pasteurellosis is one of the most common disease of sheep and other ruminants throughout the world that causes losses because of high mortality, treatment costs, reduced weight gain, delayed marketing, and unthriftiness among survivors of the flock [1,2]. *Mannheimia haemolytica*, formerly known as *Pasteurella haemolytica*, is the etiological agent of ovine pneumonic pasteurellosis or enzootic pneumonia [3,4], with the occasional involvement of *Pasteurella multocida* serotypes. They are Gram-negative coccobacilli to pleomorphic, non-motile, non-spore forming, fermentative, which may show bipolar staining [5]. *M. haemolytica* being an important primary and opportunistic pathogen of sheep is capable of causing infection in cases of compromised body defense by a variety of stress factors such as transportation, malnutrition, adverse physical, environmental or climatic conditions, previous or co-infection with certain respiratory viruses, mycoplasma or other types of bacteria, etc. [6]. The disease has economic significance for India, which is a rich source of diverse ovine germplasm with 74.5 million sheep, which is 6.813% of world sheep population [7]. There are limited published reports regarding the extent of involvement of *M. haemolytica* in pneumonia in the sheep and goat population of India [8].

Diagnosis of *M. haemolytica* has been traditionally based on clinical symptoms, isolation of the organism and extensive phenotyping and capsular serotyping, which are not only time consuming and very tedious but encounter considerable deficiencies regarding validity and reproducibility of results. For instance, culture conditions can influence the expression of phenotypic attributes thus hampering the stability and reproducibility of phenotypic methods [9,10]. In recent years, genotypic methods, especially nucleic acid based assays, allow the bacterial identification with improved sensitivity and rapidity [11].

In order to improve *M. haemolytica* detection by polymerase chain reaction (PCR), we employed multiplex PCR (mPCR). One step reaction amplifying multiple loci through mPCR is a robust and widely used tool for rapid and specific identification of pathogenic bacteria [12]. Our three-amplicons based mPCR assay targeted *P. haemolytica* serotype-1 specific antigens (PHSSA) (2) and Rpt2 genes [12] of *M. haemolytica* and a sheep-specific mitochondrial 12S ribosomal RNA (rRNA) gene [13] as a non-competitive internal amplification control (IAC). This internal PCR control provided assurance that the tissue samples were successfully amplified and detected. An mPCR based on two-amplicons targeted PHSSA and Rpt2 genes of *M. haemolytica* that enabled specific identification of *M. haemolytica* isolates in culture. The mPCR enabled rapid, accurate and direct detection of *M. haemolytica* in lung tissue of affected animals and bacterial isolates in culture.

## Materials and Methods

### Ethical approval

Ethical approval was not necessary as samples were collected from dead animals.

### Sample collection

In this study, the archived lung tissues from sheep that died of pneumonia and that had been tested for *M. haemolytica* by culture were used. Samples were collected from July 2012 to October 2012. The animals were of farms maintained under semi-intensive system in semi-arid tropical region of Rajasthan, India at a longitude of 75°-28′ E, latitude of 26°-26′ N and an altitude of 320 m above mean sea level.

### Bacterial isolation

Aseptically collected swabs from lung tissues were directly streaked onto blood agar base (Himedia, Mumbai, Maharashtra, India) supplemented with 5% defibrinated sheep blood. After aerobic incubation at 37°C for 24-48 h suspected colonies were selected for further identification [14]. Pure cultures were obtained from these isolates after studying morphological characteristic of bacterial colonies and bacteria in Gram-stained smears. The isolates were subjected to routine biochemical tests i.e. nitrate reduction, catalase, oxidase, H2S, urease and growth on MacConkey agar.

### DNA isolation

#### DNA extraction from lung tissue

Bacterial genomic DNA was isolated from all lung tissues that had already been subjected to cultural isolation. For isolation of bacterial DNA, approximately 1 g of lung tissue was homogenized in 3 ml of sterile distilled water and suspension was centrifuged at 1500 × *g*. DNA was extracted from the supernatant using QIAGEN DNeasy Blood and Tissue Kit (Qiagen, Germantown, MD, USA) as per manufacturer’s instructions. The DNA purity was checked on 0.8% agarose gel electrophoresis and stored at −20°C until used.

#### DNA extraction from bacterial isolates

A few colonies from the phenotypically characterized pure cultures of *M. haemolytica* from 24-48 h growth on blood agar plates were transferred into 1.5 ml Eppendorf tubes. The bacterial genomic DNA was extracted using QIAGEN DNeasy Blood and Tissue Kit as per manufacturer’s instructions (Qiagen, Germantown, MD, USA). The DNA purity was checked on 0.8% agarose gel electrophoresis and stored at −20°C until used.

#### Primers and PCR conditions

The oligonucleotide primers used in this study are listed in Table-1. Primers targeting PHSSA and Rpt2 genes of *M. haemolytica* were obtained from previously published work [1,12] and were synthesized commercially from Integrated DNA Technologies. A primer pair targeting the mitochondrial 12S rRNA, a housekeeping gene of sheep was designed to act as an IAC for PCRs done with DNA extracted directly from lung tissue samples. PCR carried out in a final volume of 25 µl of reaction mixture containing ×1 PCR buffer, 2 mM MgCl_2_, 200 µM dNTPs mix (Fermentas), 0.2 µM of each primers, 1.5 units of Taq DNA polymerase (Sigma) and 50 ng of DNA template (DNA from lung tissues or bacterial isolates) in a thermocycler (peqSTAR 96 Universal Gradient). PCR conditions were optimized by putting gradient PCRs with annealing temperature ranging from 45°C to 65°C. The annealing temperature of which 48°C was found to be optimum and therefore selected for the further reaction. The PCR conditions used to amplify all three gene fragments included an initial denaturation temperature of 95°C for 3 min, followed by 35 cycles each of 95°C for 1 min, 48°C for 1 min and 72°C for 30 s and a final cycle at 72°C for 5 min. A negative control consisting of all component of reaction mixture except the DNA template was included in the PCR. Positive controls were included in the mPCR from tissue samples. The PCR products were analyzed by visualization of desired size of DNA bands in the ethidium bromide stained agarose gel (2.0% w/v, 0.5X Tris borate EDTA buffer) under gel documentation system [15].

**Table-1:** The primer pairs used in PCR for *M. haemolytica*.

Target gene	Primers	Sequence (5’→3’)	Length of PCR products (bp)	Reference
Rpt2	Forward	GTTTGTAAGATATCCCATTT	1022	[12]
	Reverse	CGTTTTCCACTTGCGTGA		
PHSSA	Forward	TTCACATCTTCATCCTC	325	[1]
	Reverse	TTTTCATCCTCTTCGTC		
12S rRNA	Forward	TAACCCTTGTMCCTTTTGSATRRK	270	[13]
	Reverse	AGACTAACTTTTAAAGATACAGTGGG		

PCR=Polymerase chain reaction, PHSSA=*Pasteurella haemolytica* serotype-1 specific antigens, *M. haemolytica=Mannheimia haemolytica*, rRNA=Ribosomal RNA

### Sequencing of PCR products

For the sequencing of the gene fragments, standard PCRs were run using Pfu polymerase (Fermentas) and PCR products were resolved using 2.0% agarose gel. The specific sized bands were excised from the gel under UV light and were subsequently purified using mini elute gel extraction kit (Qiagen, Germantown, MD, USA). The purified products were sequenced by Xcelris genomics, India and compared with sequence available in the NCBI database.

## Results

### Clinical symptoms of affected animals

The cases of enzootic pneumonia occurred on a well-managed farm under a semi-intensive system in the semi-arid tropical region of Rajasthan, India. Clinically, the animals of the affected flock exhibited pyrexia and severe respiratory distress, especially in young animals (>3 months of age). The clinical course was acute and short leading to the sudden death of lambs. Following respiratory disturbances, the infected animal appeared dull, depressed, anorectic and with respiratory grunts in advanced stages of the diseases. The survived animals became chronically infected with reduced productivity.

### Post-mortem findings

An autopsy of the dead animals revealed gross pathological lesions close to the fibrinous bronchopneumonia with clear fibrinous pleurisy. The affected portions of the lungs revealed consolidation, dark red coloration and deposition of fibrinous strands. Irregular shaped areas of coagulative necrosis were frequently observed within the pneumonic portions of lung parenchyma. The mediastinal and bronchial lymph nodes were found to be congested and filled with often congested and edematous. Sometimes, respiratory tract including the trachea and major bronchi were congested and often filled with frothy fluid.

### Biochemical characteristics of the pathogen

Bacteria isolated from lung tissues of 12 sheep that died of pneumonia were identified as *M. haemolytica*. All the *M. haemolytica* were Gram-negative, coccobacilli, few unusually long rods and pleomorphic, growth on MacConkey agar, catalase positive, oxidase positive, nitrate positive, urease-negative, and H2S positive [14]. Some of the other bacterial isolates from the lung tissues were *Escherichia coli* and *Staphylococcus* spp. PCR was used for the detection of *M. haemolytica* directly from the lung tissue samples that had been tested for this organism by culture.

### Polymerase chain reaction

**Figure-1:**
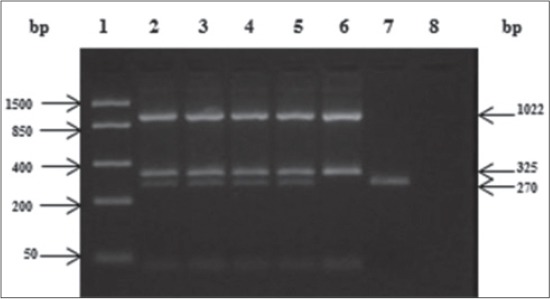
Multiplex polymerase chain reaction amplification profile of *Mannheimia haemolytica* from DNA isolated directly from lung tissue. Lane 1 - FastRuler low range DNA ladder (Fermentas # SM1103), Lane 2 to 5 - 1022 bp, 325 bp and 270 bp product of Rpt2, *Pasteurella haemolytica* serotype-1 specific antigens (PHSSA) and 12S ribosomal RNA (rRNA) gene respectively, Lane 6 - *M. haemolytica* isolate positive for Rpt2 and PHSSA gene, Lane 7- Sheep specific 12S rRNA gene as internal amplification control in lung tissue and Land 8 - negative control.

**Figure-2:**
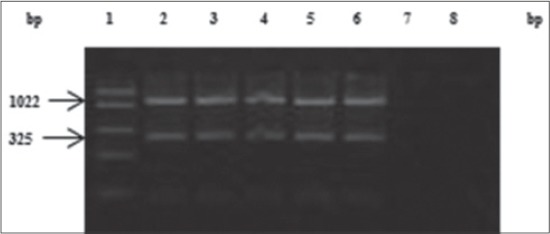
Multiplex polymerase chain reaction amplification profile Lane 1 - FastRuler low range DNA ladder (Ferments #SM1103), Lane 2 to 6 - 1022 bp product of Rpt2 and 325 pb product of *Pasteurella haemolytica* serotype-1 specific antigens gene for identification of *Mannheimia haemolytica*, Lane 7 - negative control and Lane 8 - Known negative (*Escherichia coli*).

All the 12 lung tissue samples tested for the presence of PHSSA and Rpt2 genes of *M. haemolytica* by mPCR were found to be positive (Figure-1). Amplification of 12S rRNA gene fragment as IAC was obtained with each mPCR reaction performed from DNA extracted directly from lung tissue samples (Figure-1). All the *M. haemolytica* were also positive for mPCR (Figure-2). No amplification was observed for negative control and known negative (*E. coli*) and desired amplicons were observed for known positives (Figures-1 and -2). All the three nucleotide sequences were deposited in NCBI GenBank (accession no. KJ534629, KJ534630 and KJ534631). Sequencing of the amplified products revealed the identity of 99%-100%, with published sequence of PHSSA and Rpt2 genes of *M. haemolytica* available in the NCBI database. Sheep specific mitochondrial 12S rRNA gene sequence also revealed the identity of 98% with published sequences in the NCBI database.

## Discussion

The complex interplay of predisposing factors like production stress in prevailing climatic conditions of the region (hot weather, diurnal variations etc.) and changing weather patterns might have led to the stress build up in the form of natural incidences of pneumonic *Mannheimiosis* in sheep. In a recent survey response by 126 member countries of World Organization for Animal Health (OIE), pasteurellosis emerged as one of the major disease of animals adversely impacted by climate change [16]. These clinical and gross pathological findings were consistent with published findings associated with *M. haemolytica* [4,6]. The identification of *M. haemolytica* with bacteriological methods is often difficult in some situations (antibiotic treatment, frozen material, autolytic material, co-isolated *Pasteurella* species and others). In an investigation, it was underlined that the serotyping does not represent a reliable method for identification of *M. haemolytica* as common capsular epitopes might exist in many bacterial species within the family *Pasteurellaceae* [9]. Because of limited reliability [17], unserotypable nature of approximately 10% of ruminant isolates [18] and non-availability of typing sera in most diagnostic laboratories in India, serotyping was not performed in the present study.

PCR-based methods yield high specificity and sensitivity for the detection of bacterial DNA [19]. The merits of mPCR, including internal controls, simultaneous amplification of two or more fragments, indications of template quantity and quality, greater discerning ability and less expense of time and reagents, make the technique a useful disease diagnostic tool and preferable to simultaneous uniplex PCR in numerous instances [20]. A number of uniplex PCR techniques have been adapted to multiplex amplifications for diagnosis of infectious diseases owing to its greater flexibility in experimental design. Virulence associated genes or loci represent ideal targets for accurate and rapid identification of pathogens by molecular methods. The evolving field of bacterial genomics and proteomics is expanding the choice of these gene targets to be employed for molecular diagnostics. Virulence associated genomic fragments homologous to PHSSA have been detected in many strains of *M. haemolytica* [21]. Serotype-1 specific antigen (ssa1) gene could have a pathobiological significance owing to its putative association with phenomenon of stress-precipitated commensal-to-pathogen conversion in the microbial population and suggested genetic correlation between ssa1 and leukotoxin (lkt), in genesis of pneumonic pasteurellosis Thus, PHSSA represent species-specific and virulence-associated gene of *M. haemolytica* [22]. Species-specific Rpt2 locus in *M. haemolytica* has a possible role in modulation of type III restriction-modification system [23]. Thus, Rpt2 and PHSSA genes in *M. haemolytica* make them a suitable molecular diagnostic target.

Concurrent amplification of 325 bp and 1022 bp band specific to PHSSA and Rpt2 gene respectively, from all *M. haemolytica* (n=12) isolates indicated the high specificity of our mPCR assay but also suggestive of virulence association the normally found commensal isolates of *M. haemolytica*. The 99-100% identity between the sequences of PCR amplicons and the NCBI GenBank sequence (CP004753.1, CP006619.1, M62363.1, U07788.1, CP006619.1, CP005972.1, CP006574.1, CP006573.1, etc.) for PHSSA and Rpt2 of *M. haemolytica* further affirmed high specificity. Sequence analysis of Rpt2 amplicons also showed characteristic CTGTG/CACAG pentanucleotide repeats within the 5’-end [23]. Also, the target sequence amplified with Pfu polymerase assured high fidelity in the sequence obtained from amplicons [15].

In the present study, the results of the one step mPCR assay was in complete agreement with the bacterial culture in identifying all the 12 isolates as *M. haemolytica* as well as their direct detection in the culture positive lung tissue samples. In previous studies, gene targets such as Rpt2, PomA, ssa1, lkt, gcp and 16S rRNA have been employed for identification of *M. haemolytica* showing similar results [1,12]. Previously, a PCR assay to detect *M. haemolytica* could not distinguish M. glucosida [24]. A recent study reported that the primers designed to amplify lkt detected not only lkt of *M. haemolytica* but also that of other Mannheimia species such as *Mannheimia* *glucosidal* and *Mannheimia ruminalis* [25]. In a recent study, higher detection rate of *M. haemolytica* genome by PCR in culture-negative lung tissues of bighorn sheep (*Ovis canadensis*) may be attributed to its inherent ability to detect dead or less number of organisms, which could not be detected by culture-based methods [25]. Thus, it is suggested that suspected cases of *M. haemolytica* cases must be confirmed by molecular test for correct diagnosis and for arriving at real incidence. Specificity of primers used in the present study ruled out the possibility of amplification of gene fragments of closely related Mannheimia species such as *M. glucosidal*, and *M. ruminalis* [1,12]. Inclusion of a non-competitive 12S rRNA target as IAC in our mPCR allowed the control over inhibitory factors within the samples improving reliability of the assay, the importance of which has previously been emphasized [13,26].

## Conclusion

The observations of the present study emphasized the PCR as a preferred method to conventional bacteriological methods in clinical laboratories for faster analysis of infectious diseases. In addition to its use in culture diagnostics, this mPCR may be valuable for detection of *M. haemolytica* in clinical samples from animals. The mPCR assay described here can, therefore, be extremely useful as a fast, reliable and sensitive complement to existing diagnostic tools. Moreover, it offers the opportunity to perform large-scale epidemiological studies regarding the role of *M. haemolytica* in clinical cases of pneumonia and other disease manifestations in sheep and other ruminants, thereby providing the basis for effective preventive strategies.

## Authors’ Contributions

All the authors designed the study. JK and RK conducted the study with assistance from SKD. SKD and RK revised the draft manuscript prepared by JK. All authors read and approved the final manuscript.
